# Effects of *Prunella vulgaris* labiatae extract on specific and non-specific immune responses in tilapia (*Oreochromis niloticus*)

**DOI:** 10.1186/2055-0391-56-3

**Published:** 2014-05-15

**Authors:** Kwan-Ha Park, Sanghoon Choi

**Affiliations:** Department of Aquatic Life Medicine, Kunsan National University, Gunsan, Geonbuk, 573-400 South Korea

**Keywords:** *Prunella vulgaris*, Respiratory burst activity, Lysozyme activity, Tilapia

## Abstract

We examined the effects of *Prunella vulgaris* Labiatae (*P. vulgaris* L.) on specific and non-specific immune responses of Nile tilapia, *Oreochromis niloticus*. The optimal concentration without toxicity of *P. vulgaris* was determined to 30-40 μg/ml *in vitro* and 120 μg/100 g of fish *in vivo. P. vulgaris* significantly elicited an antibody titer compared to FCA or β-glucan. β-glucan plus *P. vulgaris* group synergistically enhanced antibody production. No significant difference in antibody production was observed between *P. vulgaris* and *P. vulgaris* plus β-glucan group. A respiratory burst activity of head kidney (HK) leucocytes of tilapia administered with 300 or 500 μg *P. vulgaris* was significantly (p < 0.05) enhanced compared with the PBS-injected control group and FCA-treated group. Maximum increase in the NBT reduction value was observed in 500 μg *P. vulgaris* group but no significant difference was found between 300 and 500 μg *P. vulgaris* group. The level of serum lysozyme activity was significantly (p < 0.05) higher in the 300 and 500 μg *P. vulgaris* than 100 μg *P. vulgaris* and FCA group. The phagocytic activities of HK leucocytes from tilapia administered with 300 and 500 μg *P. vulgaris* were significantly (p < 0.05) higher than 100 μg *P. vulgaris* and the control group. *P. vulgaris* was revealed with a good immunoadjuvant evoking the specific and non-specific immune responses of tilapia.

## Background

One of the most promising methods for controlling diseases in aquaculture is strengthening the defense mechanisms of fish through prophylactic administration of immunostimulants [1]. Immunostimulants are naturally occurring compounds that modulate the immune system by increasing host resistance to infectious pathogens, and they have been widely used in aquaculture [2–4]. Traditional disease control strategies employing antibiotics and chemical disinfectants are no longer recommended due to the emergence of bacterial resistance as well as concerns over the environment and wildlife protection. Although vaccination has been shown to be an effective prophylactic method for disease control in fish [5], there are some methodological problems related to high costs and stress [6]. Already, remarkable success has been achieved using immunostimulants as a more environmentally friendly approach to disease management [7–9].

Several compounds, including β-glucans, chitin, algal and mistletoe extracts, and bacterial polysaccharides, have been used to enhance immunity and disease resistance in a variety of fish species [7, 10–12]. β-glucan administration has been reported to augment antibody production, complement activity, lysozyme activity, phagocytic activity, and respiratory bursts in channel catfish *Ictalurus punctatus*[13], Atlantic salmon *Salmo salar*[14], rainbow trout *Oncorhynchus mykiss*[15], gilthead rainbow trout *Sparus auratus*[16], and sea bass *Dicentrarchus labrax*[17].

*Prunella vulgaris* is a perennial herb that is used in traditional medicine for the clinical treatment of sore throat, fever, and accelerated wound healing [18, 19]. The organic fraction of *P. vulgaris* exhibits antioxidative and antimicrobial activities [18], whereas aqueous extracts of *P. vulgaris* inhibit HIV-1 infection [20]. In aquaculture, *P. vulgaris* was reported to have efficacy as a dietary supplement, although only up-regulation of natural immunity was observed [21]. To further study the availability of *P. vulgaris* as an immunoadjuvant to elicit a vaccination effect, we measured the specific antibody titer following intraperitoneal injection of *P. vulgaris* with an antigen in tilapia as a fish model.

To overcome the disease problem affecting fish culture systems, the present study applied indigenous *P. vulgaris* as an appropriate immnoadjuvant in order to augment specific and non-specific immunities as well as disease resistance in fish.

## Materials and methods

### Fish

Nile tilapia, *Oreochromis niloticus*, fish weighing about 100-150 g each were obtained from a fish farm in Kunsan National University, Korea and acclimated for 2 weeks to laboratory conditions in 70 L glass aquaria containing re-circulated and aerated water at 23-23°C. They were acclimated to this environment for at least 2 weeks prior to use and fed daily using a commercial diet during the adaptation and experimental periods. The health status of the animals was checked daily by observing fish behavior, and there were no clinical symptoms such as abnormal swimming patterns or body color changes.

### Reagents

Nitroblue tetrazolium (NBT), Percoll, hemocyanin (HC), 3-(4,5-dimethylthiazol-2yl)-2,5-diphenyl-2H-tetrazolium bromide (MTT), phorbol myristate acetate (PMA), and Minimum essential medium (MEM) were purchased from Sigma Chemicals CO. Hanks balanced salt solution (HBSS), fetal bovine serum (FBS), and antibiotic-antimycotic were obtained from Gibco BRL, Grand Island, NY. Sodium nitrite, sulfanylamide, and phosphoric acid were purchased from ICN Biomedicals. Bakers’ yeast and *Saccharomyces cerevisiae* purchased from Oriental Yeast Co. Ltd., Tokyo, and thioglycollate broth was obtained from Difco Laboratories, Detroit, USA.

### Extraction of *P. vulgaris*

*P. vulgaris* originating from South Korea was kindly donated by an herbal medicine company (Sanyacho-Nongwon, Namyangju, Korea). Extraction of *P.* vulgaris was performed according to Lee’s method [22]. Briefly, the chopped flowers, stems, and leaves of *P. vulgaris* (100 g) were placed in distilled water (1,000 ml) and stirred at 4°C overnight. After centrifugation at 15,000 × *g* for 20 min, the supernatant was filtered through 0.2 μm pore-sized filters. Protein content of *P. vulgaris* extract was determined using a commercial protein assay kit (Bio-Rad Lab, USA) and stored at 4°C.

### *In vitro* and *in vivo* toxicities of *P. vulgaris*

The *in vitro* toxicity of *P. vulgaris* was tested against EPC and CHSE-214 fish cell lines. Respective cell lines (1 × 10^6^ cells) were dispensed into each well of a 24-well plate (Costar, USA), followed by administration of various concentrations of *P. vulgaris* extract ranging from 10 ng to 100 μg/ml. After incubation at 24°C for 3 days, MTT assay was performed according to the method of Daly et al. [23]. Briefly, the tissue culture plates were centrifuged at 500 × g for 10 min, after which the supernatant fluids were carefully removed without disturbing the cell pellet or formazan precipitate. The formazan crystals were then dissolved by addition of 200 μl of dimethyl sulphoxide (DMSO) (Sigma) to each well, followed by 25 μl of glycine buffer (0.1 M glycine, 0.1 M NaCl, pH 10.5). Contents of the wells were then thoroughly mixed with a multichannel pipette. After 10 min, formazan development was read at 595 nm using an ELISA reader (ASYS HITECH, Austria).

To determine whether or not *P. vulgaris* has serious toxicity *in vivo*, 100 and 1000 μg of *P. vulgaris*/100 g of fish were intraperitoneally (I.P.) injected into seven fish per group. Fish blood was collected 4 days after injection, and the concentrations of glutamic oxaloacetatic trams aminase (GOT), glutamic pyruvate transaminase (GPT), and c-creatin were determined on a Fuji Dry Chem System (Fuji Photo Film Co. Ltd, Japan).

### Administration of *P. vulgaris* to elicit non-specific immune response in tilapia

Tilapia were divided into five groups of seven fishes per group. Fish in each group were I.P injected with 100, 300, and 500 μg of *P. vulgaris*/100 g of fish in 0.5 ml of phosphate-buffered saline (PBS). The remaining group of fish was injected with an equivalent volume of sterile PBS or 1:1 emulsified Freund's complete adjuvant (FCA) (Sigma) as a control. On day 4 post-injection, blood and head kidney leucocytes were obtained from each fish.

### Antibody production upon administration of *P. vulgaris* plus other immunostimulants

The immunostimulating effect of *P. vulgaris* was compared with those of FCA and β-glucan based on elevated antibody production. HC was used as an antigen to evoke a specific antibody response. For I.P. injection, 300 μg of *P. vulgaris* and 100 μg of HC were mixed and administered in a volume of 200 μl. Tilapia were divided into six groups (five fish per group), after which HC was injected alone (control) or mixed and injected with 100 μl of other adjuvants. The total injection volume was adjusted to 200 μl in all experiments. *P. vulgaris* was I.P. injected at a concentration of 300 μg suspended in 200 μl of phosphate-buffered saline (PBS). In the FCA group, 100 μl of FCA was 1:1 emulsified with HC suspended in 100 μl of PBS. In the FCA plus *P. vulgaris* group, 100 μl of FCA was added to the *P. vulgaris* and HC mixture and then I.P. injected at a volume of 200 μl. The optimized concentration (50 μg) of β-glucan was injected with HC at a volume of 200 μl. In the β-glucan plus *P. vulgaris* group, 50 μg of β-glucan and 300 μg *P. vulgaris* were mixed together and I.P. injected at a volume of 200 μl. On day 30 post-injection, blood was harvested from the fishes in each group, followed by antibody titer assay using an ELISA reader.

### Isolation of head kidney (HK) leucocytes

The method described by Santarem et al. [24] was followed with some modifications. The tilapia HK was dissected out by ventral incision, cut into small fragments, and then transferred into 5 ml of HBSS. Cell suspensions of the HK were obtained by teasing HK tissues with two slide glasses in HBSS in a Petri dish (Coring, USA). After sedimentation of tissue debris at 4°C for 1 min, the supernatants were removed. HK cell suspensions were then layered over a 34-51% Percoll gradient and centrifuged at 1000 × g for 40 min at 14°C. After centrifugation, leucocyte bands located above the 34-51% interfaces were collected using a Pasteur pipette and washed twice at 120 × g for 8 min in HBSS. The concentration of viable cells was determined by trypan blue exclusion.

### Serum

Blood was collected from the dorsal aorta of tilapia. Blood was allowed to clot at 20°C for 30 min and then cooled at 0°C for 1 h. Serum was obtained by centrifugation at 1000 × g for 8 min. Sera were frozen at -20°C until used.

### Reactive oxygen intermediates (ROI) production assay

ROI production by tilapia kidney cells after administration of *P. vulgaris* was assessed by monitoring reduction of NBT [25]. Leucocytes (1 × 10^5^ cells) were washed once with HBSS at 60 × g for 3 min at 4°C and then incubated in 100 μl of complete media in the presence of PMA and 1 μg/ml of NBT. After 1 h of incubation at 25°C, excess NBT was washed out with PBS, and the leucocytes were fixed with 70% methanol. After discarding the methanol, the leucocytes were washed twice with PBS. The reduced formazan was then solubilized with 120 μl of KOH and 140 μg of DMSO, after which optical density values were read at 620 nm on an ELISA reader.

### Lysozyme activity

Serum lysozyme activity was measured using a modified turbidimetric microtiter plate technique according to Ellis [6]. Briefly, a standard suspension of 0.15 mg/ml of *Micrococcus lysodeikticus* (Sigma) was prepared in 66 mM phosphate buffer (pH 6.0). Tilapia serum (50 μl) was then added to 1 ml of the bacterial suspension, after which the absorbance reduction was recorded at 0.5 and 4.5 min intervals at 450 nm on a spectrophotometer (SHIMADZU UV-1600PC). One unit of lysozyme activity was defined as a reduction in absorbance of 0.001/min.

### Phagocytic activity

Tilapia HK leucocytes were adjusted to 1 × 10^6^ cells/200 μl/well in 5% FBS-MEM and dispensed in an 8-well slide chamber (Nunc, Denmark), followed by overnight incubation at 25°C. Following incubation, 1 × 10^7^ cells/ml of zymosan (Sigma) was added. The mixture was incubated at 25°C for 1 h with occasional shaking, after which 50 μl of the mixture was smeared onto a glass slide, air-dried, and stained with Wright’s solution. Phagocytic activity (PA) [26] was calculated by enumerating 500 leucocytes per fish under a microscope. PA = number of cells ingesting zymosans/number of cells observed × 100.

### Statistical analysis

Statistical significance of the differences between the groups was calculated by applying Student’s 2-tailed *t*-test.

## Results and discussion

*Prunella vulgaris* is a perennial plant known for its self-healing properties in Western herbal medicine [27, 28], and it traditionally has been used for treating various diseases such as an allergies and inflammation in East Asian countries [29]. In addition, *P. vulgaris* has been reported to have immunomodulatory effects such as activation of macrophages [27, 28, 30]. The effect of *P. vulgaris* on fish immunity in aquaculture has only been reported by Harikrishnan et al. [21]. Specifically, they investigated the dietary effects of *P. vulgaris* on the non-specific immune response as well as disease resistance against *Uronema marinum*. In the present study, we tested the availability of *P. vulgaris* as a potent immunoadjuvant to achieve an enhanced vaccination effect. Furthermore, the *in vitro* and *in vivo* toxicities of *P. vulgaris* were investigated in tilapia as a fish model. Lastly, to determine the optimal amount of *P. vulgaris* extract that evokes an immune response in tilapia, we administered *P. vulgaris* extract by intraperitoneal (I.P.) injection.

**Figure 1:**
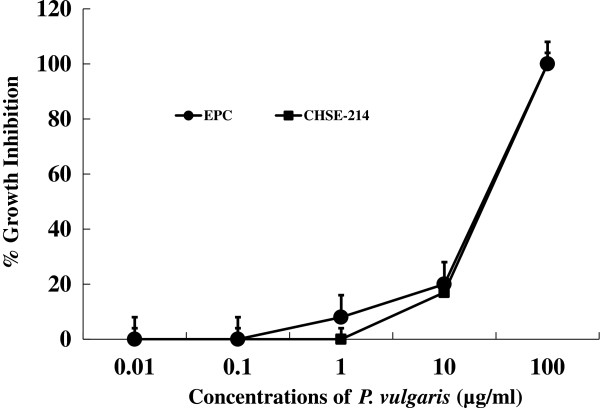
***In vitro*** **cytotoxicity of** ***P. vulgaris*** **on EPC and CHSE-214 fish cell lines.** The cells (1 × 10^6^) from each cell line were incubated with *P. vulgaris* ranging from 10 ng to 100 μg/ml and incubated at 24°C for 3 days. Formazan development was read at 595 nm using ELISA reader (ASYS HITECH, Austria). Error bars represent SD from the mean of triplicate wells. The result is a representative of three experiments.

**Figure 2:**
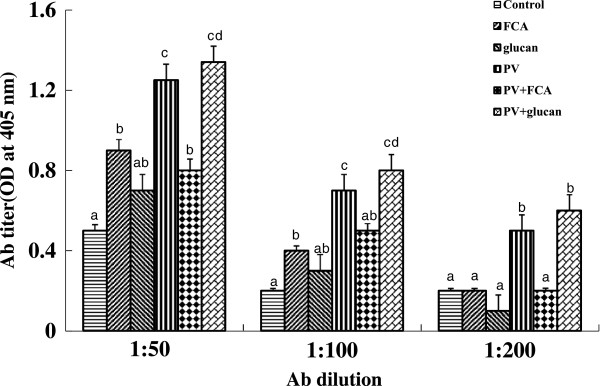
**Antibody titer induced by** ***P. vulgaris*** **administered in tilapia.** Six groups (5 fish/group) of fish were I.P. immunized with hemocyanin supplemented with *P. vulgaris* (PV), FCA, *P. vulgaris* + FCA (PV + FCA), β-glucan, *P. vulgaris* + β-glucan (PV + glucan) and PBS as a control, respectively. Data represent the mean + S.D. (n = 5). Statistical differences (p < 0.05) between groups are indicated by different letters over the bar.

First, we tested the *in vitro* and *in vivo* toxicities of *P. vulgaris* against transformed fish cell lines and tilapia. The half-killing concentrations of *P. vulgaris* against EPC and RTG-2 cells were 30 and 40 μg/ml, respectively (Figure 1). These *in vitro* toxicities were similar to a previous study in which Korean mistletoe showed negligible toxicity on mammalian cell lines [31]. Table 1 shows the levels of GOT, GPT, and c-creatin in blood from tilapia sensitized with *P. vulgaris*. There were no significant differences in toxicity between the groups administered 100 and 1000 μg of *P. vulgaris*/100 g of fish. Further, the GOT, GPT, and c-creatin levels of the PBS control group were within normal ranges, indicating non-toxicity. In contrast, a previous study showed that a 10-fold greater concentration of mistletoe injected into eel significantly augmented toxicity [11]. The immunomodulatory effect of *P. vulgaris* was compared with those of FCA and β-glucan based on HC-specific antibody production. As shown in Figure 2, *P. vulgaris* induced significantly stronger antibody production than either FCA or β-glucan. Although β-glucan failed to elicit efficient antibody production, β-glucan plus *P. vulgaris* synergistically enhanced antibody production. However, no significant difference in toxicity between the *P. vulgaris* and *P. vulgaris* plus β-glucan groups was observed, indicating that *P. vulgaris* alone has strong immunoadjuvant activity.

**Table 1:** **The levels of GOT, GPT and c-creatin in tilapia sera following injection of** ***P. vulgaris***

Standards for toxicity	Doses of ***P. vulgaris*** injected in fish		
	PBS	P100/100^1^	P1000/100
GOT	19 ± 3 mg/ML	23 ± 5 mg/ML	25 ± 3 mg/ML
GPT	40 ± 6 mg/ML	39 ± 5 mg/ML	42 ± 6 mg/ML
c-creatin	31 ± 4 mg/ML	28 ± 5 mg/ML	33 ± 7 mg/ML

^1^μg of *P. vulgaris*/g of fish.

**Figure 3:**
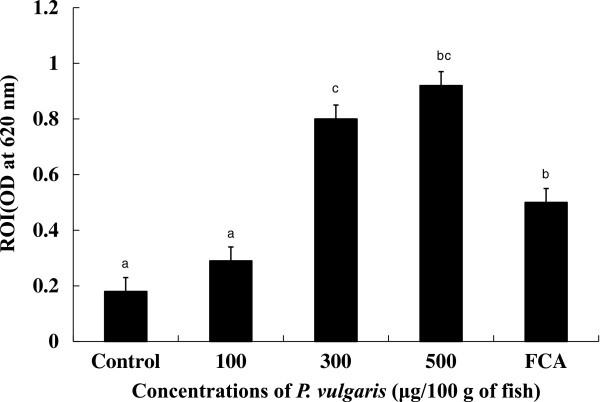
**Respiratory burst activity of head kidney leucocytes at 4 days post-injection of** ***P. vulgaris*** . Five groups (7 fish/group) of fish were I.P. injected with 0, 100, 300 and 500 μg/100 g of fish, and FCA. Data represent the mean + S.D. (n = 7). Statistical differences (p < 0.05) between groups are indicated by different letters over the bar.

Phagocytes produce respiratory bursts as a form of attack against invasive pathogens. As such, they are common measure of the defense ability against pathogens, although excessive accumulation of reactive oxygen intermediates (ROIs) is extremely toxic to host cells [32]. ROIs such as superoxide (O_2_^−^), hydrogen peroxide (H_2_O_2_), hydroxyl radical (OH), and singlet oxygen play important roles in the antimicrobial activity of phagocytic cells [33]. As shown in Figure 3, ROI production was significantly (*p* < 0.05) up-regulated in HK leucocytes from tilapia injected with 300 and 500 μg of *P. vulgaris*/100 g of fish compared to both the control and FCA groups, suggesting that ROIs are an indicator of *P. vulgaris*-induced non-specific immunity in tilapia. Although ROI production in the 100 μg of *P. vulgaris* group was higher than that in the control group, the difference was not significant. Maximum NBT reduction value was observed in the 500 μg of *P. vulgaris* group, but no significant difference was observed between the 300 and 500 μg of *P. vulgaris* groups. On the other hand, injection of more than 500 μg of *P. vulgaris*/100 g of fish failed to up-regulate ROI production (data not shown). Usually, the effect of immunostimulants is strongest at intermediate dosages with minimal activity and even toxicity at high doses [34, 35]. This phenomenon has been established in fish through *in vivo*[36, 37] and *in vitro* studies [38].

**Figure 4:**
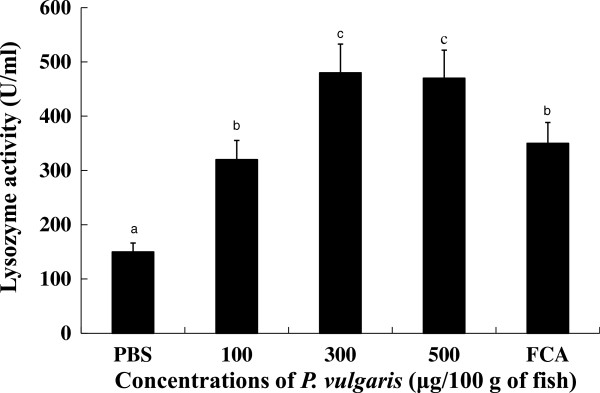
**Lysozyme activity of tilapia head kidney leucocytes at 4 days post-injection of** ***P. vulgaris*** . Five groups (7 fish/group) of fish were I.P. injected with 0, 100, 300 and 500 μg/100 g of fish, and FCA. Data represent the mean + S.D. (n = 7). Statistical differences (p < 0.05) between groups are indicated by different letters over the bar.

Lysozyme has both bactiericidal as well as opsonin effects that activate the complement system and phagocytes to prevent infection and disease [39]. Figure 4 shows lysozyme activities in the sera of tilapia with or without *P. vulgaris* administration. Serum lysozyme activities were significantly (*p* < 0.05) higher in the 300 and 500 μg of *P. vulgaris* groups compared to the 100 μg of *P. vulgaris* and FCA groups. Further, there was no significant difference in lysozyme activity between the 300 and 500 μg of *P. vulgaris* groups. Considering that serum from *P. vulgaris*-injected fish showed elevated lysozyme activity, *P. vulgaris* is likely to play a critical role in evoking lysozyme activity from tilapia kidney phagocytes. However, injection of 1000 μg of *P. vulgaris* reduced lysozyme activity compared to the 300 and 500 μg of *P. vulgaris* groups (data not shown), suggesting that an excess concentration of *P. vulgaris* interferes with lysozyme activity in tilapia.

**Figure 5:**
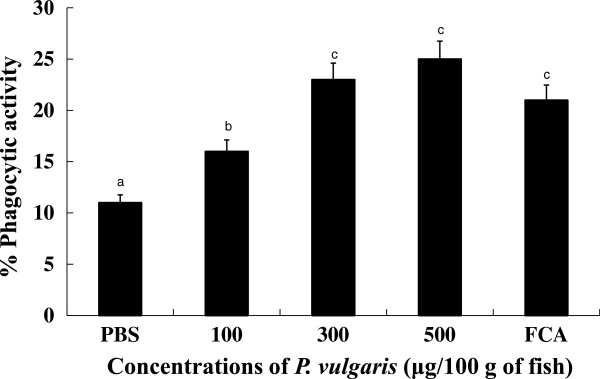
**Phagocytic activity of tilapia head kidney leucocytes at 4 days post-injection of** ***P. vulgaris*** . Fish were I.P. injected with 0, 100, 300 and 500 μg/100 g of fish, and FCA. Phagocytic activity was measured on day 4 after injection. Data represent the mean + S.D. (n = 7). Statistical differences (p < 0.05) between groups are indicated by different letters over the bar.

Phagocytes are the first cells to recognize invading foreign bodies and are thus central to cell-mediated and humoral immunities [40]. To test whether or not *P. vulgaris* can influence phagocytic activity, tilapia kidney leucocytes sensitized with *P. vulgaris* (100, 300, and 500 μg of *P. vulgaris*) were incubated overnight with zymosans. In our study, instead of foreign pathogens, zymosans were treated to phagocytes from either *P. vulgaris*-injected tilapia or non-treated tilapia. As shown in Figure 5, the phagocytic activities of HK leucocytes isolated from tilapia injected with 300 and 500 μg of *P. vulgaris* were significantly higher compared to the 100 μg of *P. vulgaris* and PBS control groups. Further, was a significant difference (*p* < 0.05) between the 100 μg of *P. vulgaris* as well as 300 or 500 μg of *P. vulgaris* groups, but no significant difference between the 300 and 500 μg of *P. vulgaris* groups themselves. Lastly, excess injection of *P. vulgaris* (1000 μg) inhibited phagocytosis and respiratory bursts in HK leucocytes isolated from tilapia.

Although the materials used in the study were different, Gopalakannan and Aurl [41] and Luo et al. [42] also reported that fish treated with a high dosage of chitosan display significantly inhibited phagocytosis compared to low dosage. This result suggests that the high level of *P. vulgaris* directly induced phagocytosis, thereby exhausting the cells.

## Conclusions

*P. vulgaris* elevated almost all non-specific immune parameters as well as specific humoral immunity. Therefore, *P. vulgaris* could be a promising immunomodulatory material for inducing specific and non-specific immune responses in fish. Further studies on using *P. vulgaris* as a dietary supplement in aquaculture are currently underway.
